# Prohibitin-1 plays a regulatory role in Leydig cell steroidogenesis

**DOI:** 10.1016/j.isci.2022.104165

**Published:** 2022-03-28

**Authors:** Geetika Bassi, Suresh Mishra

**Affiliations:** 1 Department of Physiology and Pathophysiology, College of Medicine, Faculty of Health Sciences, University of Manitoba, Winnipeg, MB R3E 3P4, Canada; 2 Department of Internal Medicine, College of Medicine, Faculty of Health Sciences, University of Manitoba, Rm. 839 JBRC, 715 McDermot Avenue, Winnipeg, MB R3E 3P4, Canada

**Keywords:** Biological sciences, Physiology, Human metabolism, Cell biology, Developmental biology

## Abstract

Mitochondria are essential for steroidogenesis. In steroidogenic cells, the initiation of steroidogenesis from cholesterol occurs on the matrix side of the inner mitochondrial membrane by the enzyme P450scc. This requires cholesterol import from the cytoplasm through the outer mitochondrial membrane, facilitated by the StAR protein. The subsequent steps leading to P450scc remain elusive. Here we report that the male transgenic mice that expressed a mutant form of a mitochondrial protein prohibitin-1 (PHB1^Tyr114Phe^) from the *Fabp-4* gene promoter displayed smaller testes, higher testosterone, and lower gonadotropin levels compared with PHB1-expressing and wild-type mice. A subsequent analysis of the testis and Leydig cells from the mice revealed that PHB1 played a previously unknown regulatory role in Leydig cell steroidogenesis. This includes a role in coordinating cell signaling, cholesterol homeostasis, and mitochondrial biology pertaining to steroidogenesis. The implications of our finding are broad as the initial stages of steroidogenesis are indistinguishable across steroidogenic cells.

## Highlights

• Tyr114Phe-PHB-1 transgenic male mice reveal PHB-1’s role in testosterone production
• PHB-1 coordinates steroidogenic signaling and events in testosterone biosynthesis
• Tyr114 residue in PHB-1 plays a regulatory role in testosterone production

## Graphical abstract

**Figure:**
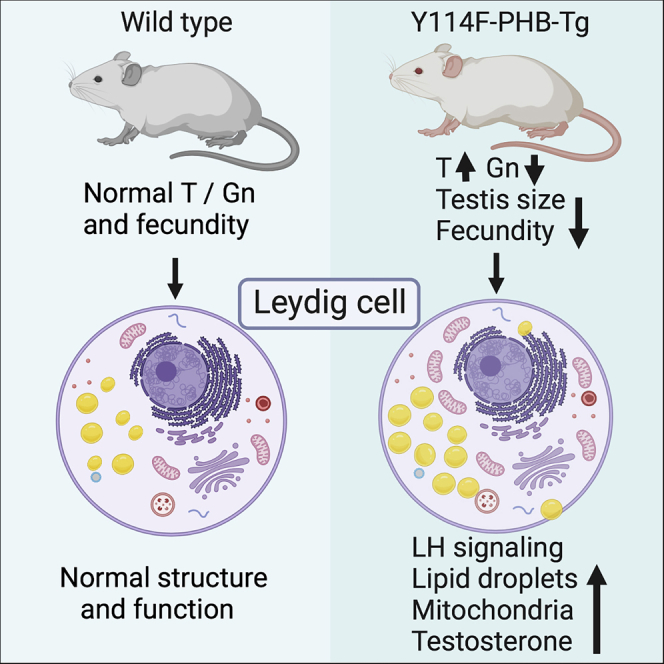

## Introduction

Steroid hormones are essential to life, as they regulate critical phases of development, such as puberty and reproductive ability (Wood et al., 2019). Altered levels of steroid hormones are associated with various pathological conditions, including infertility (Reichman and Rosenwaks, 2017), metabolic and immune dysregulation (Faulkner and Belin de Chantemèle, 2019), as well as hormone-dependent cancers (Rodriguez-Gonzalez et al., 2008). Thus, steroid hormone biosynthesis is finely regulated to not only ensure adequate amounts are produced, but also to avoid hormone insufficiency or excess. In this context, the feedback relationship between the different trophic hormones and the respective steroid hormones (e.g., ACTH-glucocorticoids, LH-testosterone, and LH/FSH-estradiol) is well established (Oyola and Handa, 2017; Kaprara and Huhtaniemi, 2018). The trophic hormone-induced cellular events in steroidogenic cells leading to steroid hormone production span different cellular compartments (i.e., the cytoplasm, mitochondria, and smooth endoplasmic reticulum) (Miller and Auchus, 2011). This arrangement is likely to facilitate a fine coordination between steroidogenic events in the different cellular compartments to control hormone levels within a normal physiological range. However, our knowledge of intracellular regulatory factors and mechanisms that may coordinate steroidogenic processes between different cellular compartments to maintain steroidogenic homeostasis (i.e., avoid hormone deficiency or excess) in steroidogenic cells remains limited. This knowledge is important because of the inherent relationship between the regulation of trophic hormones at the hypothalamus-pituitary level and the potential pathological consequences of their dysregulation in steroidogenic glands through altered tropic hormones, and, consequently, in the body at large, owing to dysregulated steroidogenesis. For example, a perturbed steroidogenesis in the adrenal glands and the testis may lead to pathologies related to chronic high or low levels of trophic hormones owing to a dysregulated negative feedback loop at the hypothalamus-pituitary level, such as congenital adrenal hyperplasia and hypogonadotropic hypogonadism, respectively.

The fundamental framework of steroid hormone biosynthesis across major steroidogenic tissues is very similar, especially within mitochondrial steps that are indistinguishable (Miller and Auchus, 2011). For example, cholesterol is the common substrate for all steroid hormones, and its transport by the steroidogenic acute regulatory protein (StAR) to the mitochondria, and the subsequent utilization by the cytochrome P450 side chain cleavage (P450scc, encoded by the *CYP11A1* gene) enzyme for the initiation of steroidogenesis, is a key step in all steroidogenic tissues (Monté et al., 1998). However, the identity of the mitochondrial protein(s) that couple the StAR function at the cytoplasmic side of the outer mitochondrial membrane (OMM) with the P450scc enzyme at the matrix side of the inner mitochondrial membrane (IMM), and the mechanisms involved, remain elusive. In addition, emerging evidence suggests that autophagy and lipophagy in Leydig cells play a role in intracellular cholesterol homeostasis and maintenance of testosterone production (Gao et al., 2018; Ma et al., 2018). Moreover, Leydig cell steroidogenesis is regulated by structural and functional changes in mitochondria (Duarte et al., 2014; Park et al., 2019). Mitochondrial fusion and fission, collectively known as mitochondrial dynamics, have been reported to play a role in steroidogenesis (Duarte et al., 2012; Wasilewski et al., 2012). In Leydig cells, hormonal stimulation triggers mitochondrial fusion through the upregulation of the fusion protein mitofusin 2, a process that is essential for steroid hormone production (Castillo et al., 2015). Thus, the mitochondria are not merely a site for the initiation of steroidogenesis involving the conversion of cholesterol into pregnenolone, but also appear to play a multifaceted role in steroidogenesis, including cholesterol proportioning and in the maintenance of cholesterol and steroidogenic homeostasis (Midzak and Papadopoulos, 2016). Thus, a better understanding of the interplay between different steroidogenic cell-specific functions (e.g., cholesterol homeostasis and mitochondrial attributes) is key to improve our understanding of this fundamental biological process in Leydig cells and other steroidogenic cell types.

Over the last 15 years, we have been interested in elucidating the role and regulation of an evolutionarily conserved, but so far poorly characterized protein, prohibitin-1 (PHB1) (Ande et al., 2012, 2014, 2016a, 2016b). For instance, we have identified the Tyr^114^ residue in PHB1 as an important phosphorylation site in relation to membrane signaling (e.g., PI3K-Akt and MAPK-ERK; Ande et al., 2009, 2012; Ande and Mishra, 2009), demonstrated its regulatory role in cell signaling, and discovered PHB1’s role in adipogenesis and lipid homeostasis (Ande et al., 2012, 2014), which have been further confirmed by others (Kang et al., 2013; Kim et al., 2013). Recently, we have shown that the transgenic mice overexpressing of PHB1 or mutant PHB (PHB1^Tyr114Phe^ or mPHB1) from the fatty acid binding protein-4 (*Fabp4*) gene promoter (for an adipocyte-specific expression) develops obesity, which is mediated through upregulating mitochondrial biogenesis in adipocytes (Ande et al., 2014, 2016a, 2016b). Unexpectedly, during their phenotypic characterization, we found that the mPHB1 mice have smaller testes but higher serum testosterone levels. Further investigation revealed that the PHB1 and mPHB1 mice overexpress PHB1 in their Leydig cells, which is consistent with a recent report showing that Fabp4 is expressed in Leydig cells (O’Hara et al., 2015). However, PHB1’s role in Leydig cell steroidogenesis is virtually unknown in the current literature. This prompted us to investigate the role of PHB1 in Leydig cell biology. We found that PHB1 is an important steroidogenic target gene in Leydig cells and plays a regulatory role in Leydig cell steroidogenesis, including cell signaling, cholesterol homeostasis, and mitochondrial biology involved in steroidogenesis. This requires the Tyr^114^ residue in PHB1, and its substitution with a phenylalanine (Phe) residue leads to the upregulation of testosterone production. Thus, we now report a previously unknown role of PHB1 in regulating interconnected steroidogenic events in different cellular compartments in Leydig cells. The implications of our findings are broad as the fundamentals of steroidogenesis, such as cholesterol homeostasis, cholesterol transport to the mitochondria, and the initiation of steroidogenesis, are common among all steroidogenic tissues.

## Results

### Male mPHB1 mice display smaller testes, elevated serum testosterone, and lower gonadotropin levels

**Figure 1:**
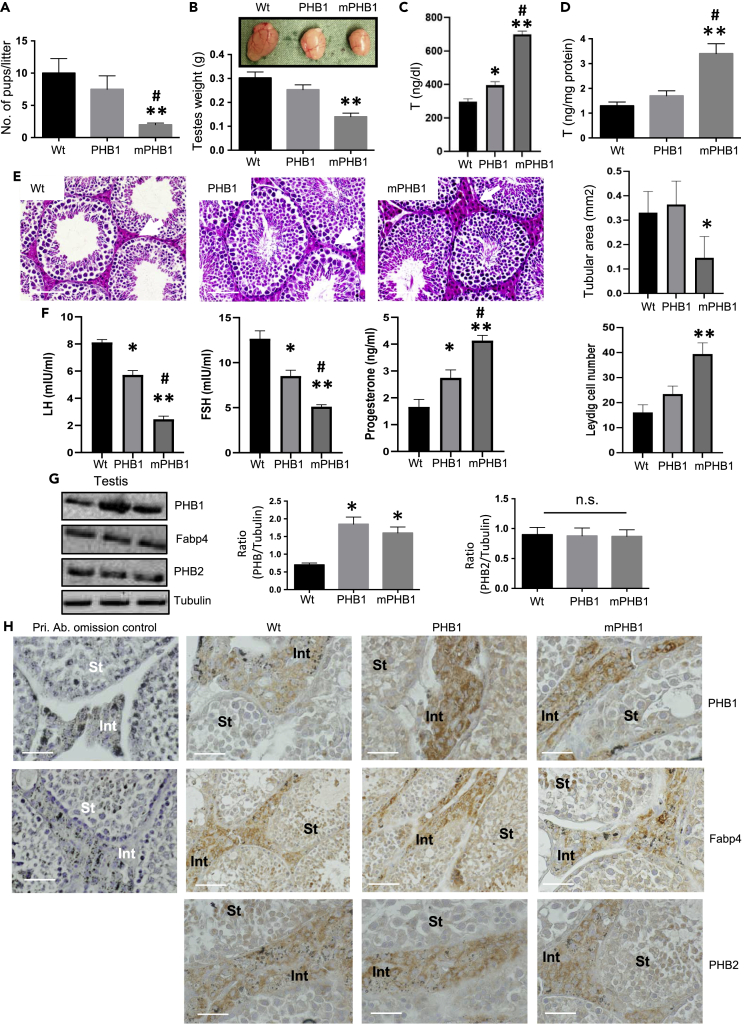
Male mPHB1 mice display reduced fertility, smaller testis, elevated testosterone, and lower gonadotropin levels (A) Histograms showing the litter size at birth in wild-type (wt) dams when sired with PHB1, mPHB1, and wt mice separately at six months of age. (B) Photographs showing testis dissected from PHB1, mPHB1, and wt mice at six months of age (upper panel). Histograms showing the quantification of testis weight from PHB1, mPHB1, and wt mice (lower panel). (C) Histograms showing serum testosterone levels in PHB1, mPHB1, and wt mice at six months of age. (D) Histograms showing testosterone production from primary Leydig cells derived from four-month-old PHB1, mPHB1, and wt mice in response to hCG (20 ng/ml). (E) Photomicrographs showing H&E-stained testis sections from PHB1, mPHB1, and wt mice at six months of age. Quantification of seminiferous tubular area (right upper panel) and Leydig cell number in testicular interstitium (right lower panel) from transgenic and wild type-mice are shown by histograms. Scale bar = 20 μm. (F) Histograms depicting serum gonadotropin levels in PHB1, mPHB1, and wt mice at six months of age. (G) Immunoblots showing PHB1, PHB2, and Fabp4 expression levels in testis from mice at four months of age. Quantification of PHB1 and PHB2 band densities are shown with histograms. Tubulin blot is included as a loading control. (H) Photomicrographs depicting immunohistochemical analysis of PHBs and Fabp-4 in the testis from PHB1, mPHB1, and wt mice at 4 months of age. The omission of primary antibodies is included as negative controls. Tissue sections were counterstained with hematoxylin for better visualization. Scale bar = 10 μm ∗*p* < 0.05, ∗∗*p* < 0.01 between wt and PHB1 or mPHB1 mice, #*p* < 0.05 between PHB1 and mPHB1 mice as determined by an ANOVA with Dunnett’s test. Data are presented as mean ± SEM (*n* = 6 mice/group or experiments repeated at least three times, as applicable). n.s., not significant, Pri. Ab., primary antibody, wt, wild type.

The immunometabolic phenotype of the PHB1 and mPHB1 mice have been described previously (Ande et al., 2014, 2016a, 2016b). During follow-up studies pertaining to these mouse models, we found a reduction in the siring ability of the male mPHB1 mice compared with age-matched, PHB1 and wild-type mice, which was significantly lower in the male mPHB1 mice by 6 months of age Figure 1A. A similar trend was also observed in the male PHB1 mice; however, the difference between PHB1 and wild-type was not significant Figure 1A. Moreover, a clear size difference in testes was observed between the male mPHB1 mice and wild-type mice, which was significantly smaller in the mPHB1 mice (∼50% reduction in the testis weight) Figure 1B. A similar trend in testis size was also observed in the PHB1 mice; however, the difference was not significant when compared with the wild-type mice (Figure 1B). In addition, the testis from the mPHB1 mice was also significantly smaller (∼35% reduction) than the testis from the PHB1 mice (Figure 1B) despite comparable body weights (Ande et al., 2014, 2016a, 2016b). However, we did not find such a difference in the size and weight of the secondary sex organs between the male transgenic and wild-type mice (not shown). This prompted us to examine serum testosterone levels within each mouse genotype. Surprisingly, serum testosterone levels were significantly higher in the mPHB1 mice compared with the age-matched PHB1 mice and wild-type mice (Figure 1C). A significant difference in testosterone levels was also found between the mPHB1 and PHB1 mice, which was higher in the former (Figure 1C). To determine whether the higher testosterone levels in mPHB1 mice was owing to its increased production from Leydig cells, we measured the testosterone production from the primary Leydig cells isolated from each mouse genotype in response to the human chorionic gonadotropin (hCG, a surrogate for the luteinizing hormone (LH) as both bind to the same LH receptors and elicit a similar response) (Lei et al., 2001). Consistent with serum testosterone levels, the Leydig cells from the mPHB1 mice produced significantly more testosterone compared with the Leydig cells from the PHB1 and wild-type mice (Figure 1D). In addition, a similar trend was found in testosterone production by Leydig cells between the PHB1 and wild-type mice (Figure 1D). In aggregate, these data suggest that PHB1 plays a regulatory role in testosterone production that involves the Tyr^114^ phosphorylation site because its substitution by a non-phosphorylable phenylalanine (Phe) residue in mPHB1 leads to increased testosterone production.

To get insight into why the differences in testis size and serum testosterone levels between the PHB1, mPHB1, and wild-type mice were occurring, we performed a histological analysis. Consistent with the testis size, the area of seminiferous tubules was significantly smaller with relatively narrow lumen and reduced intra-tubular germ cell contents in mPHB1 mice compared with PHB1 and wild-type mice (Figure 1E). However, the interstitial space between seminiferous tubules were relatively larger and wider in the testis from mPHB1 mice, with increased Leydig cell population (Figure 1E), indicating a potential relationship between the Leydig cells and higher testosterone levels in the mPHB1 mice.

Next, we sought to uncover serum gonadotropin levels in mPHB1 and PHB1 mice because of its role in the regulation of testis structure and functions, as well as its feedback relationship with testosterone (Miller and Auchus, 2011). Both LH and FSH levels were significantly lower in mPHB1 mice compared with the PHB1 and wild-type mice (Figure 1F), suggesting a potential negative feedback inhibition by higher testosterone levels in the mPHB mice. A significant difference in gonadotropin levels were also observed between the PHB1 and wild-type mice, which was lower in PHB1 mice (Figure 1F). Collectively, a more pronounced hypogonadotropic hypergonadism phenotype of the mPHB1 mice when compared to the PHB1 mice would suggest that PHB1 plays a regulatory role in testosterone production by Leydig cells, which involves the Tyr^114^ residue in PHB1.

### PHB1 and mPHB1 mice overexpress PHB1 in their Leydig cells

The *Fabp-4* gene promoter that we used to develop the PHB1 and mPHB1 transgenic mice models is often used for adipocyte-specific gene manipulation because it is primarily expressed in adipocytes (Kusminski et al., 2012). However, an unexpected testicular phenotype of the mPHB1 mice and increased testosterone production from the primary Leydig cells isolated from them raised a question about the potential expression of *Fabp-4* in the testis/Leydig cells, and, consequently, an overexpression of PHB1 or mPHB1 within them, contributing to a testicular phenotype, as observed in the PHB1 and mPHB1 mice (Figures 1A–1D). Subsequent research revealed that Fabp-4 was recently reported to express in the testis in mice, specifically in Leydig cells (O’Hara et al., 2015). To find out if this is the case for PHB1 transgenic mice, we first analyzed testis samples from them using immunoblotting and immunohistochemistry. Fabp-4 protein was detected in the testis by both methods (Figures 1G and 1H) and Leydig cell-specific expression of Fabp-4 was apparent from immunohistochemical analysis (Figure 1H). Importantly, an increased expression of PHB1 was found in the testis samples using immunohistochemistry, especially in Leydig cells in the testicular interstitium of both transgenic mouse models compared with wild-type mice, matching the expression pattern of Fabp-4 (Figure 1H). In addition, we examined the expression levels of PHB1’s homologous protein, PHB2, which is known to form heterodimers with PHB1 in mitochondria. No difference in PHB2 protein levels were found in the testis samples from the PHB1-Tg and mPHB1-Tg mice compared with the wild-type mice (Figures 1G and 1H). Together, this evidence confirmed that the PHB1 and mPHB1 mice overexpress PHB1 in their Leydig cells.

### PHB1 is an LH-regulated protein and plays a role in hormone production by Leydig cells

**Figure 2:**
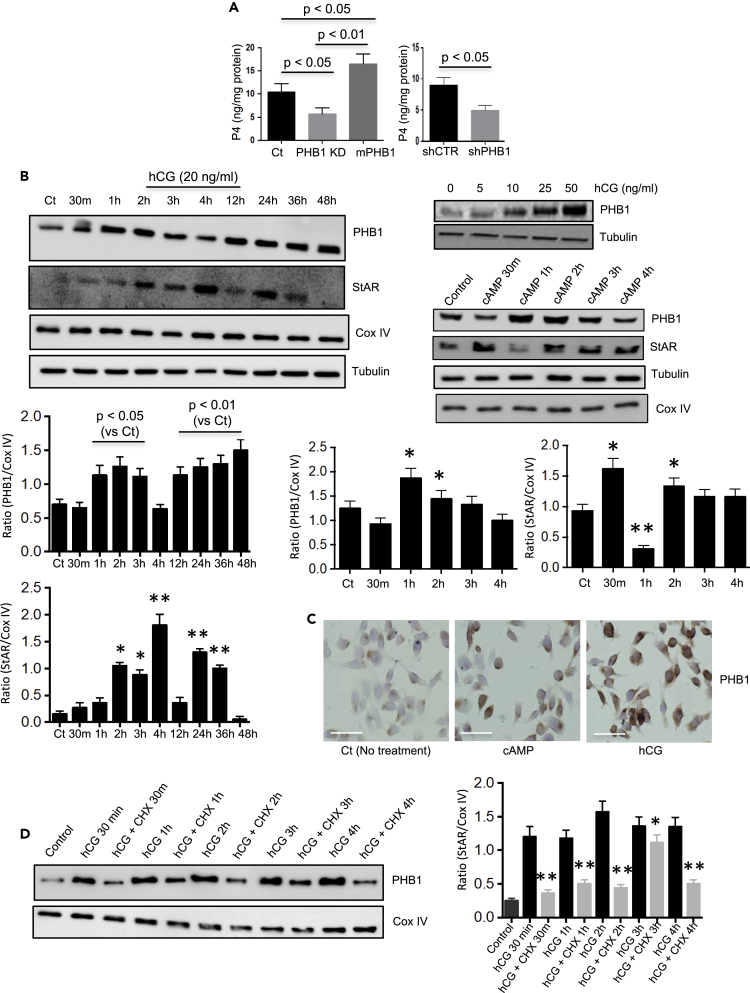
PHB1 is an LH-regulated protein and plays a role in hormone production by Leydig cells (A) Histograms showing the effect of CRISPR/Cas9- (left panel) and shRNA-mediated (right panel) PHB1 knockdown on hCG-induced progesterone production from MA-10 cells. (B) Immunoblots showing time- and dose-dependent effects of hCG stimulation on PHB1 levels in MA-10 cells (left and upper right panel). Dose-dependent effect of db-cAMP on PHB1 levels in MA-10 cells (middle right panel). Cox-IV blot is shown as a loading control. Quantification of band intensities are shown with histograms (lower panel). Data are presented as mean ± SEM (*n* = 3). ∗*p* < 0.05, ∗∗*p* < 0.01 between Ct and cAMP or hCG stimulation (as applicable) as determined using an ANOVA with Dunnett’s test. (C) Photomicrographs showing immunocytochemical analysis of PHB1 levels in MA-10 cells in response to db-cAMP and hCG. Scale bar = 10 μm. (D) Representative immunoblots showing the effect of cycloheximide (CHX) treatment on hCG-induced changes in PHB1 levels in MA-10 cells. Cox-IV blot is shown as a loading control. ∗*p* < 0.05, ∗∗*p* < 0.01 between hCG and hCG + CHX by Dunnett’s test. Data are presented as mean ± SEM (*n* = 3).

To further explore PHB1’s role in Leydig cell steroidogenesis, we resorted to MA-10 cells, a model murine Leydig cell line, which produces progesterone (P4) as a major product instead of testosterone (Ascoli, 1981). A CRISPR/Cas9-mediated Phb1 knockdown performed in MA-10 (CRISPR/Cs9-Phb-MA-10) cells significantly decreased hCG-induced P4 production in comparison with control MA-10 cells (Figure 2A), whereas the overexpression of mPHB1 in CRISPR/Cs9-Phb-MA-10 cells not only rescued P4 production, but further enhanced in comparison with control group (Figure 2A). A similar effect of shRNA-mediated Phb1 knockdown on steroidogenesis was also observed in MA-10 cells in response to hCG stimulation (Figure 2A). A similar outcome using two different experimental approaches would mean that the observed effect was specific to the manipulation of Phb1 levels in MA-10 cells. This finding prompted us to investigate whether PHB1 is a target gene for LH in Leydig cells, as LH plays a central role in almost every aspect of LC steroidogenesis (Medar et al., 2021). The stimulation of LCs with hCG led to the upregulation of PHB1 protein in a time- and dose-dependent manner and displayed both acute (within 2 h) and chronic (36–48 h) effects (Figure 2B). To further confirm acute regulation of PHB1 protein in Leydig cells, we repeated the experiment with dibutyryl cyclic-AMP (db-cAMP or cAMP). A similar effect on PHB1 levels was observed in response to db-cAMP (Figure 2B). Thus, in our subsequent experiments, we used only db-cAMP (when reasonable) for consistency and to avoid batch variation in hCG preparations. An acute upregulation of PHB1 protein in response to hCG and db-cAMP during a 1- to 2-h period (Figure 2B) but not in mitochondrial protein Cox IV level would imply that an augmented level of PHB1 is not owing to an augmented number of mitochondria. This finding indicates a similarity with the regulation of StAR levels, which is known to acutely regulated in steroidogenic cells (Bose et al., 2002), including Leydig cells (Duarte et al., 2014; Castillo et al., 2015). Thus, we examined StAR levels using immunoblotting. Interestingly, a relationship was observed between PHB and StAR levels in response to hCG stimulation, which was inversely related during the first hour, at 4 and 48 h of the treatment, and then both proteins showed similar pattern during other time points (Figure 2B). Moreover, in response to db-cAMP, a dynamic change in StAR doublet bands was observed during the first four hours of stimulation showing a correlation with PHB1 protein levels to some extent (Figure 2B). In addition, we performed immunocytochemical analysis of PHB1 level in MA-10 cells in response to hCG and db-cAMP stimulation. Again, the upregulation of PHB1 levels in response to steroidogenic stimulation was apparent in MA-10 cells (Figure 2C). Our finding of acute regulation of PHB during first few hours of hCG stimulation led us to investigate whether PHB1 is regulated at the translational level in Leydig cells. For this, we stimulated MA-10 cells with hCG in the presence of cycloheximide (CHX), a protein synthesis inhibitor. A reduction in the expression level of PHB1 was observed (Figure 2D), confirming that PHB1 is regulated at the translational level in Leydig cells. Collectively, these data confirmed that PHB1 is a gonadotropin-regulated protein in Leydig cells and plays a role in steroid hormone production by Leydig cells.

### Leydig cells from PHB1 and mPHB1 mice showed distinct mitochondrial and lipid droplet characteristics

**Figure 3:**
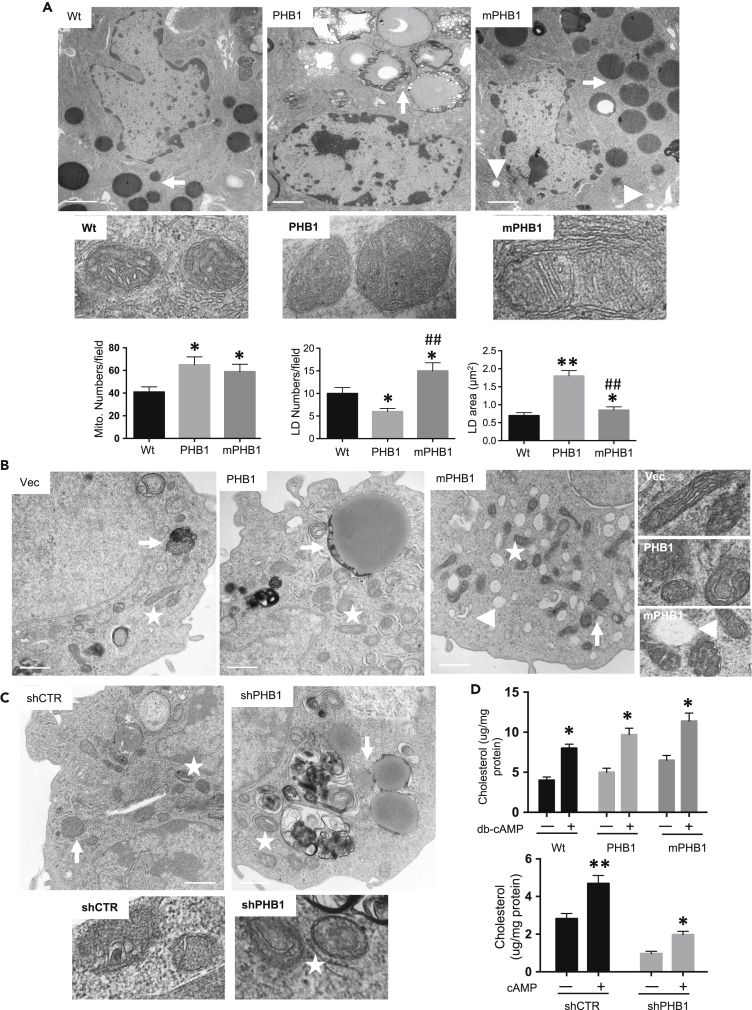
Leydig cells from PHB1 and mPHB1 mice showed distinct mitochondrial and lipid droplet characteristics (A) Photomicrographs depicting TEM analysis of Leydig cells in the testis from PHB1, mPHB1, and wt mice (upper panel, Scale bar = 500 nm) and magnified view of their mitochondria (middle panel). Histograms depicting mitochondrial number, as well as and lipid droplet size and number in Leydig cells (lower panel). ∗*p* < 0.05, ∗∗*p* < 0.01 between wt and PHB1 or mPHB1 mice, #*p* < 0.05, ##*p* < 0.01 between PHB1 and mPHB1 mice as determined using an ANOVA with Dunnett’s test. Data are presented as mean ± SEM (*n* = 6). (B and C) Photomicrographs depicting TEM analysis of PHB1 manipulated MA-10 cells (Scale bar = 500 nm) and magnified view of their mitochondria (right panel in B and lower panel in C) are also shown for better visualization. (D) Histograms showing cholesterol levels in Leydig cells from PHB1, mPHB1, and wt mice (upper panel) and in PHB1 manipulated MA-10 cells (lower panel) in response to db-cAMP stimulation. ∗*p* < 0.05, ∗∗*p* < 0.01 between stimulated and unstimulated cells by Student’s *t* test. Data are presented as mean ± SEM (*n* = 3 mice/group or experiment repeated three time, as applicable). Star indicates mitochondria, arrowhead indicates lysosomes, and arrow indicated lipid droplets. LD, lipid droplets; shCTR, scramble control shRNA; shPHB1, PHB1 shRNA.

There is much evidence in the previous literature, suggesting that PHB1 plays a wide-ranging and interconnected role in mitochondrial biology and lipid homeostasis (Merkwirth et al., 2008; Osman et al., 2009a, 2009b). For example, we have shown that transgenic mice overexpressing PHB1 in adipocytes develop obesity, which involves mitochondrial biogenesis (Ande et al., 2014), and others have reported that PHB1 has a role to play in mitochondrial phospho-lipid homeostasis in different model organisms (Merkwirth et al., 2008; Osman et al., 2009a, 2009b). Furthermore, the PHB1 family member MEC-2 binds cholesterol in relation to membrane signaling and functions (Huber et al., 2006), whereas Erlin-1 and Erlin-2 are highly enriched in the detergent-soluble ER fraction in a cholesterol-dependent manner (Browman et al., 2006). Thus, a possibility exists that PHB1 and mPHB1 may influence mitochondrial biology and cholesterol homeostasis within Leydig cells, and this could be the reason for the increased serum testosterone levels observed in the PHB1 transgenic mice (Figure 1B) and primary Leydig cells derived from them (Figure 1D). Therefore, we analyzed testis samples from the PHB1 transgenic mice using transmission electron microscopy (TEM). A substantial increase in lipid droplets (LDs) was observed in Leydig cells from both the PHB1 and mPHB1 mice compared with the wild-type mice (Figure 3A). However, a difference in the size, number, and morphology of the LDs were apparent in Leydig cells between them (Figure 3A). In Leydig cells of the PHB1 mice, the LDs were relatively larger, irregular in shape, and showed signs of lysosomal degradation, whereas Leydig cells from the mPHB1 mice displayed relatively smaller, uniform, and regular LDs, indicating a difference in lipid processing (Figure 3A). In addition, a difference in mitochondrial shape was observed between the PHB1 and mPHB1 mice, which were primarily oval or circular in Leydig cells from the PHB1 mice, whereas they appeared fusiform or elongated in Leydig cells from mPHB1 mice (Figure 3A). Moreover, a difference in mitochondrial cristae were noticeable, which were more prominent in Leydig cells from the mPHB1 mice compared with the PHB1 mice (Figure 3A). In addition to structural differences, an increase in mitochondrial density was found in Leydig cells from the PHB1 and mPHB1 mice compared with the wild-type mice (Figure 3A). Moreover, a similar change in LDs and mitochondrial features were observed in PHB1 and mPHB1-overexpressing MA-10 cells (Figure 3B). In addition, a substantial increase in lysosome population was observed in mPHB1 expressing MA-10 cells (Figure 3B). Importantly, performing a PHB1 knockdown in MA-10 cells led to the dysregulation of mitochondrial structure (e.g., fragmentation of mitochondrial cristae) and LDs (Figure 3C). Collectively, these data indicate that PHB1 and mPHB1 have different effects on lipid/cholesterol handling, mitochondrial structure, and functions in Leydig cells, which may have direct effects at the mitochondrial level, or perhaps indirect effects owing to changes in upstream signaling events and impaired lipid handling.

Next, we measured cholesterol levels in primary Leydig cells isolated from the testis samples of the PHB1 and mPHB1 mice. Cholesterol levels were found to be significantly higher in Leydig cells from both the PHB1 and mPHB1 mice when compared with the wild type mice under a basal state; however, the difference between the PHB1 mice and mPHB1 mice was not significant (Figure 3D). In response to cAMP stimulation, a significant increase in cholesterol levels was observed in all groups in comparison with the unstimulated respective control groups (Figure 3D). However, the amplitude of increase was maximum in Leydig cells from the mPHB1 mice and minimum in Leydig cells from the wild-type mice (Figure 3D), suggesting that Leydig cells from the mPHB1 mice are more efficient in cholesterol uptake in response to steroidogenic stimulation. Importantly, shRNA-mediated knockdown of PHB1 in MA-10 cells led to a significant decrease in cholesterol uptake under basal and stimulated condition in comparison with scramble shRNA transfected control cells (Figure 3D). Together, these data suggest that PHB plays a role in cholesterol uptake and handling in Leydig cells.

### PHB1 contains putative cholesterol binding motifs and interacts with cholesterol

**Figure 4:**
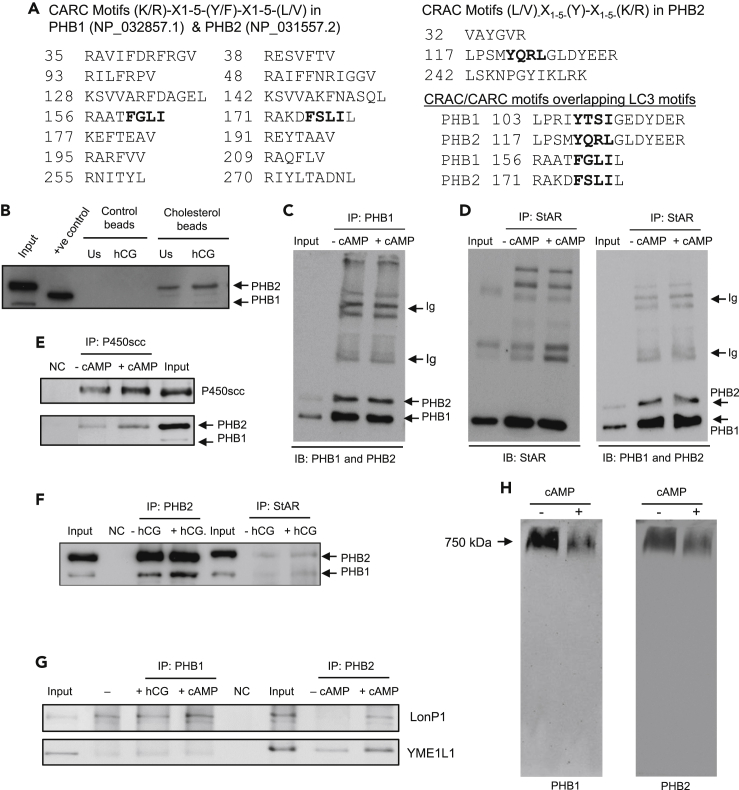
PHB1 contains putative cholesterol binding motifs, interacts with StAR and P450scc, and forms heterodimeric megacomplex with PHB2 in Leydig cells (A) Multiple PHB1 and PHB2 peptide sequences containing putative CRAC and CARC motifs. Overlapping LC3 binding motif in PHB1 and PHB2 are shown in bold. (B) Immunoblots depicting PHB1 pulldown using cholesterol immobilized agarose beads. Only agarose beads (without cholesterol) wereincluded as negative control and recombinant PHB1^28−272^ as a positive control (*n* = 3). (C) Immunoblots depicting co-immunoprecipitation of PHB1 heterodimeric partner PHB2 from MA-10 cells (−/+ db-cAMP treatment, 0.5 mM) as determined by the respective protein-specific antibody. Only a small amount (∼1/5th) was used in input lane to avoid band saturation and potential spill over to the adjacent lane. (D) Immunoblots showing co-immunoprecipitation of PHBs with StAR from MA-10 cells (−/+ db-cAMP stimulation, 0.5 mM). Again, only a small amount (∼1/5th) was used in the input lane. (E) Immunoblots showing co-immunoprecipitation of PHBs with P450scc from MA-10 cells with or without db-cAMP treatment (0.5 mM). (F) Immunoblots showing co-immunoprecipitation of PHB1 with PHB2 from MA-10 cells with or without hCG stimulation (20 ng/mL). (G) Immunoblots showing co-immunoprecipitation of mitochondrial proteases with PHB1 and PHB2 from MA-10 cells with or without hCG (20 ng/mL) or db-cAMP treatment (0.5 mM). (H) Immunoblots showing heterodimeric megacomplex of PHB1 and PHB2 in MA-10 cells with or without db-cAMP treatment, as determined by BN-PAGE and immunoblotting. All experiments were repeated for at least three times. IB, immunoblotting; IP, immunoprecipitation; Ig, immunoglobulin band; NC, negative control.

A potential link between PHB1 and lipid/cholesterol homeostasis in Leydig cells (as revealed by TEM analysis and cholesterol uptake in PHB1-manipulated MA-10 cells) prompted us to examine PHB1’s protein sequence for putative cholesterol binding domains or motifs (Romanowski et al., 2002; Yang et al., 2014). An analysis of PHB1’s protein sequence using the NCBI BLAST tool displayed that PHB1 lacks the cholesterol-binding domain present in the StAR family of proteins; however, a number of putative Cholesterol Recognition Amino Acid Consensus sequences (generally referred to as the CRAC motif) and inverted CRAC sequences (generally referred to as the CARC motif) were identified (Figure 4A). Both PHB1 and its heterodimeric partner PHB2 were found to contain multiple cholesterol binding motifs, including motifs spanning the conserved tyrosine residues (Figure 4A). To determine whether putative cholesterol-binding motifs in PHB1 interacts with cholesterol, we performed a cholesterol-binding assay using cholesterol-immobilized beads and MA-10 cell lysates (with/without hCG stimulation). Both PHB1 and PHB2 were successfully pulled down by the cholesterol beads but evaded the control beads (Figure 4B). These data suggest that PHB1 and PHB2 contain multiple cholesterol-binding motifs, which appears to interact with and bind cholesterol.

### PHB1 interacts with StAR and P450scc and forms a heterodimeric megacomplex with PHB2 in Leydig cells

PHB1 and its homologous protein PHB2 form a heterodimeric megacomplex spanning the inner mitochondrial membrane (Tatsuta et al., 2005) (where P450scc resides) and have been identified as phosphoproteins along with StAR in rat granulosa cells (a steroidogenic cell type in the ovary) (Thompson et al., 1997). Thus, PHB1 heterodimeric megacomplex may potentially interact with proteins involved in cholesterol transport and utilization across mitochondrial membranes (e.g., StAR and P450scc). To explore this possibility, we immunoprecipitated PHB1 from cAMP-stimulated and unstimulated MA-10 cell lysates using protein-specific monoclonal antibodies and Dynabeads. As expected, PHB2 was co-immunoprecipitated with PHB1 (Figure 4C). Next, we immunoprecipitated StAR and P450scc using a protein-specific antibody. Both PHB1 and PHB2 were co-immunoprecipitated with StAR (Figure 4D), whereas only PHB2 was found to be co-immunoprecipitated with P450scc (Figure 4E). Interestingly, PHB2 was co-immunoprecipitated with both P450scc and StAR (Figures 4D and 4E). Thus, we immunoprecipitated PHB2 similarly and analyzed the results using immunoblotting. Only PHB1 was co-immunoprecipitated with PHB2 (Figure 4F). As our findings are suggestive of an inverse and dynamic relationship between StAR and PHB1 protein levels during their acute regulation (Figure 2B), we examined the potential interaction of mitochondrial proteases with PHB1 and PHB2 because they are known to interact or regulate them (Merkwirth et al., 2008; Osman et al., 2009a, 2009b). Mitochondrial protease LonP1 and YME1L-1 were co-immunoprecipitated with PHBs, and their band intensities were relatively higher under db-cAMP-stimulated conditions (Figure 4G), suggesting the potential interactions between mitochondrial proteases and PHBs in the acute regulation of StAR.

To confirm whether PHB1 and PHB2 form a heterodimeric megacomplex in Leydig cells, we analyzed cell lysates prepared from db-cAMP-stimulated and unstimulated cells using BN-PAGE and immunoblotting. The heterodimeric complex of PHBs was detected by both protein-specific antibodies, confirming their formation in MA-10 cells (Figure 4G). Notably, the band density of the megacomplex was found to be relatively less intense under stimulated conditions (Figure 4H), indicating a potential importance of the dynamics of the heterodimeric complex and their interaction with other partners in Leydig cells in response to steroidogenic stimulation. Collectively, this data suggests that the PHB1 and PHB2 heterodimers interact with StAR, mitochondrial proteases, and P450 in MA-10 cells.

### PHB1 modulates PKA and ERK signaling in Leydig cells

**Figure 5:**
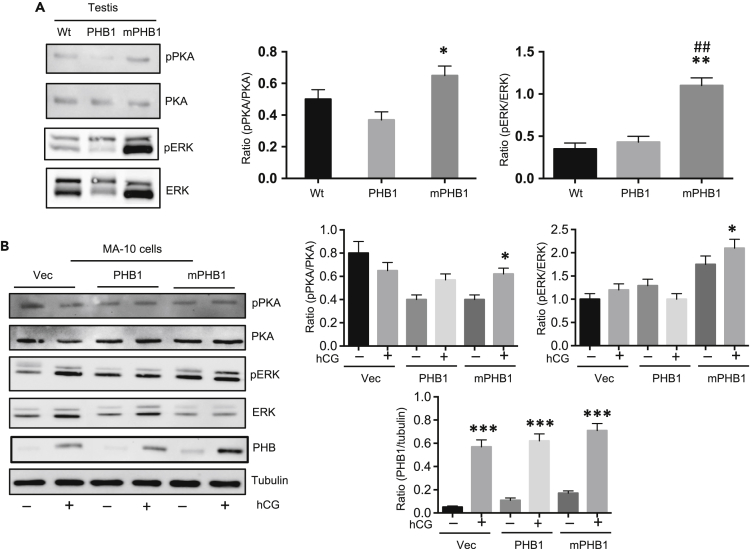
PHB1 modulates PKA and ERK signaling in Leydig cells (A) Immunoblots showing pPKA and pERK levels in the testis from PHB1, mPHB1, and wt mice (left panel). Quantification of protein band densities are shown by histograms (middle and right panels). ∗*p* < 0.05, ∗∗*p* < 0.01 between wt and PHB1 or mPHB1 mice, ##*p* < 0.01 between PHB1 and mPHB1 mice, as determined using ANOVA with Dunnett’s test. Data are presented as mean ± SEM (*n* = 3). (B) Immunoblots showing pPKA and pERK levels in MA-10 cells (with or without hCG stimulation) transfected with different PHB1 constructs (left panel). Vector only transfected cells were used as a control. Cox-IV blot is shown as a loading control. Histograms showing quantification of protein band densities (right panel). ∗*p* < 0.05, ∗∗∗*p* < 0.001 between unstimulated and stimulated cells in each experimental group using Student’s *t* test. Data are presented as mean ± SEM (*n* = 3).

Previously, we discovered the phosphorylation of PHB1 at the Tyr^114^ residue occurs in relation to insulin signaling (Ande et al., 2009; Ande and Mishra, 2009), and have shown its importance in adipocyte differentiation, including a modulatory role in MAPK-ERK signaling in a context-dependent manner (Ande et al., 2012). Moreover, works by others have shown that PHB1 undergoes phosphorylation at the Tyr^114^ residue in many cell types in relation to growth factors (IGF, EGF) (Rajalingam et al., 2005), hormones (FSH) (Chowdhury et al., 2013) and diverse immune signaling pathways (Ande et al., 2016a, 2016b; Kim et al., 2013). Thus, we examined the activation level of cell-signaling pathways (i.e., cAMP-PKA and MAPK-ERK) in the testis samples taken from the PHB1-Tg and mPHB1-Tg mice. As the cAMP-PKA signaling pathway plays a central role in mediating the LH response in Leydig cells (Medar et al., 2021), we first investigated the phospho-PKA (p-PKA) in testicular lysates by immunoblotting using a phospho-specific antibody. A relatively higher p-PKA level was found in the testis samples from the mPHB1-Tg mice compared with the PHB1-Tg and wild-type mice (Figure 5A). In addition to PKA, the MAPK-ERK pathway plays a role in LH signaling in Leydig cells (Duarte et al., 2014). As PHB1 modulates MAPK-ERK signaling, we determined the phospho-ERK (p-ERK) levels in the testis samples from the PHB1-Tg and mPHB1-Tg mice by immunoblotting using a phospho-specific antibody. The p-ERK level was found to be significantly upregulated in the mPHB1-expressing testis samples compared with the PHB1-expressing and control testis samples from the wild-type mice (Figure 5A). However, such a difference in p-ERK levels was not found between the testis from the PHB-overexpressing and wild-type mice (Figure 5A). These data indicate that PHB1 plays a regulatory role in the regulation of basal cAMP-PKA and MAPK-ERK signaling in the testis/Leydig cells, which require the Tyr^114^ residue in PHB1, as its substitution for mPHB1 leads to increased p-ERK levels. Moreover, the effect mPHB1 on p-ERK was relatively more apparent than the p-PKA levels (Figure 5A). To further validate PHB1’s role in the modulation of ERK phosphorylation, we transfected CRISPR/Cas9-Phb-MA-10 cells with different PHB1 constructs and examined the effect of hCG stimulation on pERK levels. Again, an increased pERK level was observed in MA-10 cells expressing mPHB1 in comparison with PHB1 and only vector-transfected cells (Figure 5B) confirming PHB1’s role in the regulation of pERK levels. Although a similar trend in p-PKA level was observed between PHB1 and mPHB1-expressing cells, the effect was not apparent like p-ERK levels (Figure 5B).

**Figure 6:**
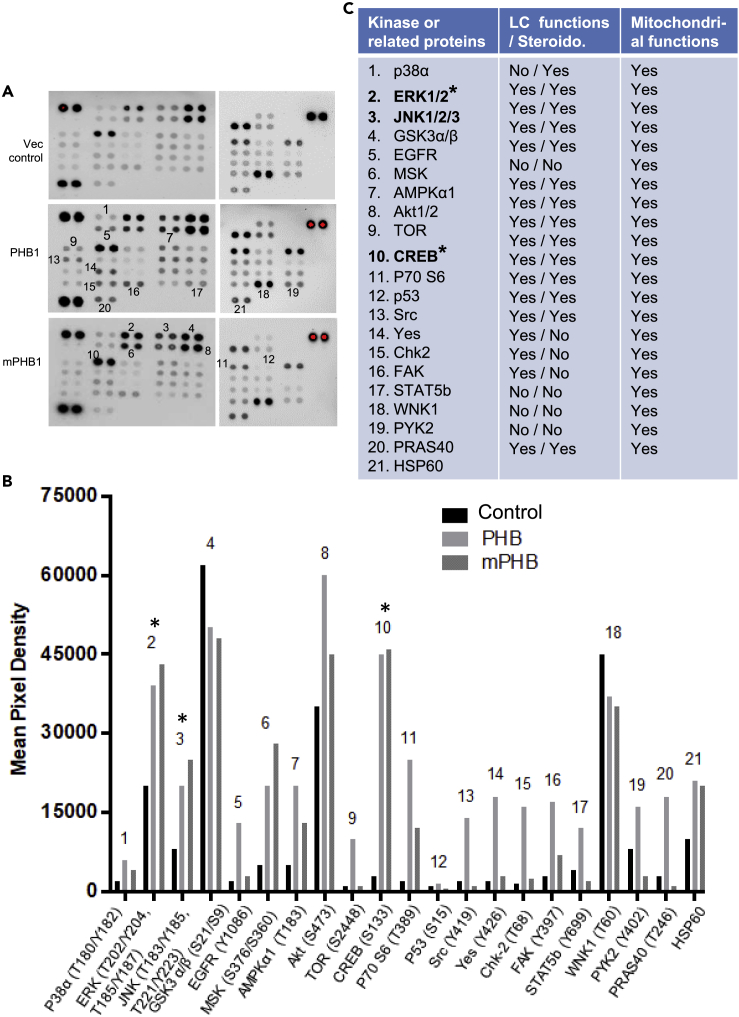
Phosphokinase array profiling of PHB1 and mPHB1 transfected MA-10 cells in response to hCG (A) Phosphokinase array blots showing differential phosphorylation levels of various signaling molecules in MA-10 cells overexpressing PHB1 and mPHB1 in response to hCG stimulation (20 ng/ml for 30 m). Vector-only transfected cells were used as a control. (B) Histograms showing comparison of signals on different arrays depicting relative change in phosphorylated kinase proteins between different experimental groups. (C) List of kinase or related proteins identified by Phosphokinase arrays and their status in relation to Leydig cell (LC) function, steroidogenesis, and mitochondrial functions based on the current literature (PubMed). Steroido, steroidogenesis. ∗ indicates mediators of PKA and MAPK signaling pathways.

**Figure 7:**
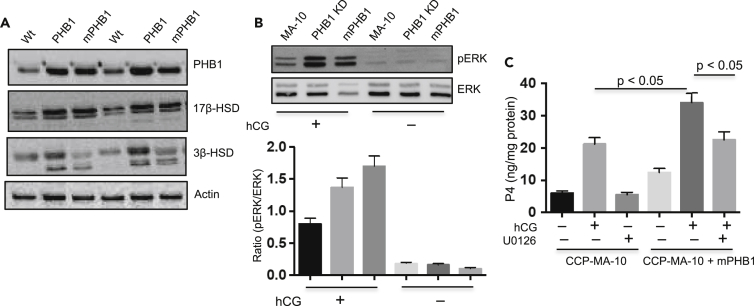
Steroidogenic markers are upregulated in the testis from PHB1 transgenic mice and PHB1 modulates ERK phosphorylation in a context-dependent manner (A) Immunoblots showing the expression levels of steroidogenic marker proteins in the testis from wild-type and transgenic mice (*n* = 3). (B) Upper panel: immunoblots depicting pERK levels in PHB1 manipulated MA-10 cells in response to hCG. Lower panel: histograms depicting quantification of band intensities (*n* = 3). The graphs represent means, and error bars indicate the standard error of the mean (SEM). (C) Histograms showing the effect of MAPK/ERK inhibitor (U0126, 7 nM)) on mPHB1-induced P4 production from CRISPR/Cs9-Phb-MA-10 (CCP-MA-10) cells in response to hCG (*n* = 3). The graphs represent means, and error bars indicate the standard error of the mean (SEM).

Next, we used a Phospho Kinase Array (containing 43 kinase phosphorylation sites and 2 related proteins – cAMP response element binding protein (CREB) and p53) to get a snapshot of various kinases and cell signaling molecules in response to db-cAMP in PHB1 and mPHB1 expressing MA-10 cells. A total of 19 signaling molecules were found to be upregulated and 2 (i.e., JNK and WNK1 kinases) were downregulated in PHB1/mPHB1 overexpressing cells when compared with the control group (Figures 6A and 6B). Interestingly, 10 of them were differentially altered in PHB1 and mPHB1 expressing cells and most of them were substantially downregulated in mPHB1 when compared with PHB1 expressing cells (Figures 6A and 6B). As expected, out of 21 altered kinase phosphorylation, 16 are known to play a role in Leydig cells, 14 in steroidogenesis, and all of them are known to be involved in the mitochondrial biology (Figure 6C), suggesting that PHB plays an important role in cell signaling and mitochondrial biology in Leydig cell pertaining to steroidogenesis. Notably, cAMP-regulated transcription factor CREB and mediators of the MAPK pathway (e.g., ERK and JNK), which plays a critical role in gonadotropin-induced chronic steroidogenesis, were found to be substantially upregulated in PHB1 and mPHB1-overexpressing MA-10 cells (Figures 6A and 6B). Thus, we examined the expression levels of steroidogenic marker proteins in the testis samples from the PHB1 and mPHB1 mice. As hypothesized, steroidogenic marker protein (3β-HSD and 17β-HSD) levels were significantly higher in transgenic mice sample compared with wild-type mice (Figure 7A), which further support our findings of higher testosterone levels in PHB1 transgenic mice. In addition, a consistent upregulation of the MAPK-ERK pathway in the testis samples and PHB1/mPHB1 manipulated MA-10 cells would imply its role in the effect of mPHB1 in Leydig cells and testicular phenotype in mPHB1-Tg mice. Thus, we investigated the effect of PHB1 knockdown on hCG-induced ERK1/2 phosphorylation in MA-10 cells. The pERK1/2 level was found to be significantly higher in PHB1 knockdown and mPHB1 expressing cells compared with control MA-10 cells (Figure 7B), further supporting our conclusion that PHB1 plays a context-dependent regulatory role in Leydig cells under basal and steroidogenic induction. This finding prompted us to explore the role of pERK in mediating mPHB1-induced enhanced steroidogenesis in Leydig cells. Incubation of mPHB1 expressing CRISPR/Cs9-Phb-MA-10 cells with MAPK/ERK inhibitor (U0126) reversed mPHB1-induced P4 production in response to hCG (Figure 7C), suggesting its role in mPHB1-induced upregulation of steroidogenesis in Leydig cells.

## Discussion

This study reports that an evolutionarily conserved pleiotropic protein named PHB1 plays a multifaceted role in Leydig cell steroidogenesis, spanning the cytosolic and the mitochondrial compartments. This includes a role in hormone-induced cell signaling, intracellular cholesterol homeostasis, and the functional coupling of StAR and P450scc across the mitochondrial membrane. A higher testosterone level in the mPHB1 mice compared with the PHB1 and wild-type mice, and a similar finding from Leydig cells isolated from them, as well as in PHB1/mPHB1-manipulated MA-10 cells implies that PHB1 plays a regulatory role in Leydig cell steroidogenesis, which involves the Tyr^114^ residue in PHB1, as its substitution in mPHB1 leads to increased steroidogenesis and consequently reduced gonadotropin levels. The mPHB1-related increased steroidogenesis in Leydig cells appears to involve augmented pERK signaling, intracellular cholesterol handling, and mitochondrial attributes. Moreover, our findings indicate that a coordination between cell signaling, and mitochondrial functions is necessary in controlling steroidogenesis in Leydig cells (i.e., to prevent steroid insufficiency or excess), which have effects on the negative feedback regulation of gonadotropin at the pituitary level.

Of note, PHB1 has been reported to play a role in ovarian granulosa cells. However, the focus of these studies were on granulosa cell proliferation, differentiation, survival, and atresia/apoptosis rather than steroidogenesis (Chowdhury et al., 2007, 2013, 2016; Thompson et al., 2004; Wang et al., 2013a, 2013b), likely because of the existing knowledge of PHB1’s context-dependent role in cell proliferation, survival, and apoptosis in different cell types. For example, Chowdhury et al. (2013) have reported that the administration of equine chorionic gonadotropin (eCG) increases PHB1 expression in the ovarian follicles and GC, but not in theca-interstitial cells within the pre-antral follicles. This increased expression of PHB1 corresponded with follicular growth, and decreased after the ovulatory LH surge and during follicular atresia. This finding would imply that the LH surge during the ovarian cycle may negatively regulate PHB1 expression. Moreover, a change in the phosphorylation levels of PHB1 and increased trafficking to the mitochondria was observed. Notably, the PHB1 phosphorylation sites under these culture conditions in response to FSH and testosterone were the Tyr^249^, Thr^258^, and Tyr^259^ sites (Chowdhury et al., 2007, 2016), which we have reported in relation to insulin signaling and lipid binding/metabolism (Ande et al., 2009, 2012, 2014, 2016a, 2016b; Ande and Mishra, 2009). Intriguingly, Wang et al. (2013b) have reported an inhibitory effect on granulosa cell steroidogenesis, whereas Chowdhury et al. (2007, 2013, 2016) have reported the opposite, which may be owing to the stage-dependent role of PHB1 in granulosa cell biology and would require further investigations. Taken together, a possibility exists that the phosphorylation of PHB1 may play a role in steroidogenic cells in response to hormones and growth factors (e.g., trophic hormones, insulin, IGF, and EGF), which are known to stimulate steroidogenesis. In summary, PHB1 possesses many features that may potentially contribute to steroidogenesis in different steroidogenic cell types.

Cholesterol serves as an essential substrate for all steroid hormones and its trafficking and homeostasis in steroidogenic cells are tightly regulated (Elustondo et al., 2017; Rone et al., 2009). Because of a highly hydrophobic chemical property, the cellular uptake and intracellular trafficking of cholesterol is mediated through different proteins (Rone et al., 2009). Proteins that interact with cholesterol often contain cholesterol-binding domains or motifs (Romanowski et al., 2002; Yang et al., 2014). For example, the StAR family members contain the START (StAR-related lipid-transfer) domain, which binds hydrophobic lipids (Tsujishita and Hurley, 2000), whereas P450scc contains the CRAC and CARC short linear motifs (Midzak et al., 2011). The central tyrosine residue in the CRAC motif (L/V-X1-5-Y-X1-5-K/R) is crucial for cholesterol binding (Elustondo et al., 2017; Rone et al., 2009). The CARC motif (K/R-X1-5-Y/F-X1-5-L/V) is similar to the CRAC motif but exhibits the opposite orientation along the polypeptide chain from the N-terminus to the C-terminus (Elustondo et al., 2017; Rone et al., 2009). In addition to the reverse orientation, CARC is distinct from CRAC in that the central aromatic amino acid can be either Tyr or Phe (Rone et al., 2009). Thus, it is possible that PHB1^Tyr114Phe^ may retain the cholesterol-binding function of PHB1 and contribute to differences in LD characteristics and steroid hormone production, as observed in Leydig cells from the PHB1 and mPHB1 mice. Moreover, previously, we have reported that Tyr^114^ in PHB is a part of other lipid-binding motifs (Ande and Mishra, 2009), and other PHB1 family members have been reported to bind cholesterol and be involved in lipid homeostasis (Merkwirth et al., 2008; Osman et al., 2009a, 2009b), including mitochondrial phospholipid metabolism (Merkwirth et al., 2008; Osman et al., 2009a, 2009b). Thus, a difference in LD characteristics in Leydig cells from the PHB1 and *m*-PHB1 mice, as revealed by TEM and their relationship with steroid hormone production, would imply that the Tyr^114^ residue plays a role in it. As LDs in Leydig cells primarily contain cholesterol and cholesterol esters, this would mean that Leydig cells from the PHB1 and mPHB1 mice differ in cholesterol handling, which may include uptake, storage, transport, and their subsequent utilization for steroidogenesis. Of note, the ultrastructural features of Leydig cells from the PHB1 mice were very similar to previous reports from *StAR* (Ishii et al., 2002) and *Cyp11A1* knockout mice (Chien et al., 2003), such as increased lipid accumulation and signs of LD degradation. However, unlike *StAR* and *Cyp11A1* knockout mice, as well as according to a histopathology of naturally occurring mutations in the StAR gene in humans (Miller, 1997), the PHB1 and mPHB1 mice do not display high trophic hormone levels. Thus, the dysregulation of lipid/cholesterol homeostasis in Leydig cells from the PHB1 and mPHB1 mice is likely owing to Leydig cell-specific alterations independent of their LH levels, which is substantially lower in comparison with the wild-type mice. It is possible that the overexpression of PHB1/mPHB1 in Leydig cells of the PHB1/mPHB1 mice leads to a change in Leydig cell-specific attributes involved in cholesterol handling, including uptake, storage, mobilization, and utilization. However, a difference in testosterone production between the PHB1 and mPHB1 mice may be owing to the upregulation of a mitochondria-specific function of PHB1 involving Tyr^114^, which is independent of its role in pERK signaling. Thus, Tyr^114^’s regulatory role may involve membrane signaling or mitochondrial functions, or a combination of both. Of note, the cholesterol binding motifs in PHB1 and the recently identified LC3 binding motifs (Wei et al., 2017) overlap with each other. This raises the important question of whether these two features of PHB1 work in a mutually exclusive and context-dependent manner to maintain cholesterol homeostasis for Leydig cell steroidogenesis in different conditions. For example, under cholesterol insufficiency, PHB1 may facilitate autophagy/mitophagy to recycle intracellular cholesterol (to maintain steroidogenesis), whereas cholesterol sufficiency and its binding to PHB1 may inhibit PHB1-mediated autophagy/mitophagy. Our finding of changes in the activation levels of an inhibitor of autophagy (i.e., WNK1 kinase; Gallolu Kankanamalage et al., 2016) in PHB1 manipulated MA-10 cells further support our hypothesis that PHB1’s role in Leydig cell steroidogenesis might involve autophagy or related mitophagy. Moreover, as PHB1 plays an important role in Leydig cell steroidogenesis, the loss of mitochondrial PHB1 owing to autophagy/mitophagy may be compensated by upregulation of PHB1 under cholesterol deficiency because PHB1 is a cholesterol sensitive gene and is upregulated under cholesterol deficiency (Dong et al., 2010). Moreover, emerging evidence suggests that lipophagy in Leydig cell plays a role in cholesterol homeostasis and testosterone production (Ma et al., 2018) and lipid homeostasis in other cell types (Liu and Czaja, 2013). The sign of lipophagy as observed in our TEM analysis of the testis samples from the PHB1-Tg mice and in PHB1-manipulated MA-10 cells would mean that PHB1’s role in testosterone production might in part be mediated through autophagy, mitophagy, or lipophagy-related cholesterol homeostasis. In addition, lipophagy may protect Leydig cells from the toxic effects of increased lipid accumulation on mitochondrial functions. Thus, PHB1 may function in multiple ways in Leydig cell biology in a context-dependent manner, as observed in the regulation of pERK1/2 levels. The implications of our research findings are broad, particularly in relation to cholesterol homeostasis in steroidogenic cells and mitochondria, as well as in the regulation of autophagy, lipophagy, and mitophagy in steroidogenic cells, adipocytes, cardiomyocytes, and hepatocytes.

The role StAR plays in the OMM, and the role P450scc plays in the IMM, in the initiation of Leydig cell steroidogenesis is well established. However, our knowledge of factors involved in their functional coupling across the outer and inner mitochondrial membranes remain limited, including mechanisms involved in cholesterol transport by StAR and its delivery to P450scc. Our co-immunoprecipitation data suggest that the mitochondrial heterodimeric complex of PHBs interacts with StAR and P450scc, and PHB1 knockdown inhibits cholesterol transport/homeostasis and P4 production in MA-10 cells. Together, this finding (along with the identification of functional cholesterol binding motifs) suggests that PHB1 plays a role in the functional coupling of StAR and P450scc during steroidogenesis in Leydig cells.

StAR protein levels are known to be acutely regulated in steroidogenic cells in response to trophic hormones (Bose et al., 2002), which remain unclear. An inverse relationship between StAR and PHB1 protein levels in PHB1 manipulated Leydig cells indicate that PHB1 may be related to cellular mechanisms involved in the acute regulation of StAR. In this context, it is important to note that PHB1 interacts with *m*-AAA and other mitochondrial proteases (Anderson et al., 2020; Steglich et al., 1999), which may be involved in this relationship. Our finding of co-immunoprecipitation of mitochondrial proteases LonP1 and YME-1L1 with PHBs in MA-10 cells support this possibility. However, unlike StAR, the basal PHB1 levels are retained in the absence of hCG or db-cAMP stimulation, which make sense in the light of the mitochondrial housekeeping functions of PHB1. To the best of our knowledge, such an acute regulation of PHB1 protein levels has not been reported in any cell type. It would be interesting to know whether PHB1 is regulated similarly in other steroidogenic cell types.

It is interesting to learn that a ubiquitous mitochondrial protein with protein and lipid scaffold properties interacts with cell type-specific proteins (e.g., StAR and P450scc) and produces cell type-specific functions. This finding is consistent with our hypothesis that the mitochondrial attributes of PHB1 contribute to the cell type-specific functions of PHB1 (in addition to its mitochondrial housekeeping functions) (Ande et al., 2016a, 2016b) and further supports our conclusion that PHB1 plays a multifaceted regulatory role in Leydig cell steroidogenesis. In addition, our findings provide a tip-off on how a single protein may function at different levels in cell biology, from a cell-neutral mitochondrial housekeeping function to the cell type-specific functions of mitochondria. Thus, this demonstrates an additional means of creating diversity (owing to cellular and functional compartmentalization) from a relatively limited number of genes and proteins, including, but not limited to, RNA splicing, PTMs, and protein domains.

The scope of our research findings is broad, and may have implications beyond Leydig cell steroidogenesis, such as with regards to corticosteroids and ovarian steroid production. Moreover, the potential health benefits and applications of our findings are wide-ranging. For instance, this new knowledge could possibly be utilized for the development of therapies to treat steroid hormone abnormalities and related comorbidities, and improving the health of elderly patients by slowing down the decline in muscle and bone health with aging in men, as well as changes in fat distribution (post menopause) and the associated health risks in women. It is likely that these forays will stimulate further investigations to unravel the vital role of PHB1 in steroidogenesis and would lead to important clinical implications.

### Conclusions

We propose that PHB1 playing a regulatory role in p-ERK signaling (mediated by Tyr^114^) provides a check and balance in coordinating membrane signaling and mitochondrial attributes pertaining to steroidogenesis to prevent the excess production of testosterone and consequently protect the hypothalamic-pituitary-gonadal axis. This function is lost in the mPHB1 mice, resulting in increased p-ERK level, testosterone production, and decreased gonadotropin levels, which remain under control in the PHB1 mice owing to normal p-ERK signaling. In addition, a similar trend in testosterone production in the PHB1 mice may be attributed to the enhanced mitochondrial functions of PHB1, independent of its role in cell signaling.

In conclusion, as our current understanding of the initial stages of steroidogenesis regarding cholesterol transport and delivery to the P450scc enzyme is still limited, we anticipate that our discovery of a novel inner mitochondrial membrane protein PHB1 will significantly add to the understanding of this elusive topic in this field. It is likely that PHB1 may have a similar role in ACTH-induced steroidogenesis in the adrenal glands and LH-induced steroidogenesis in the ovary. Moreover, our findings point toward a coordination between intracellular compartment-specific events in controlling steroidogenesis that warrants further investigation.

### Limitation of the study

We have only used mouse models and murine cell lines. However, this should not be a limiting factor owing to the general importance of our research finding because the fundamental aspect of steroidogenesis (e.g., cholesterol homeostasis in steroidogenic cells and the functional coupling of StAR and P450scc across the mitochondrial membranes for the initiation of steroidogenesis), which is the main finding of this study, is conserved in mice and humans. For example, the perturbation of StAR functions in the mouse models mimics the same phenotype observed owing to genetic disorders in humans (Miller and Auchus, 2011).

## STAR★Methods

### Key resources table

**Table:** 

REAGENT or RESOURCE	SOURCE	IDENTIFIER
**Antibodies**		

Anti-PHB1	Cell Signaling Technology	Cat#2426S
Anti-PHB2	Cell Signaling Technology	Cat#14085S
Anti-StAR	Cell Signaling Technology	Cat#8449S
Anti-Cyp11A1	Cell Signaling Technology	Cat#14217S
Anti-Total ERK	Cell Signaling Technology	Cat#9102S
Anti-Phospho ERK	Cell Signaling Technology	Cat#4695S
Anti-Total PKA	Cell Signaling Technology	Cat#4782S
Anti-Phospho PKA	Cell Signaling Technology	Cat#5661S
Anti-FABP4	Cell Signaling Technology	Cat#2120S
Anti-LonP1	Cell Signaling Technology	Cat#28020S
Anti-YME1L1	Proteintech Group	Cat#11510-1-AP
Anti-Tubulin	Cell Signaling Technology	Cat#2128S
Anti-Rabbit IgG HRP linked	Cell Signaling Technology	Cat#7074S

**Bacterial and virus strains**		

XL-5	Origene Technologies	Ref# 25
PHB	Origene Technologies	Ref# 25
Y114FmPHB	Origene Technologies	Ref# 25
PHB1shRNA	Dharmacon Inc.	RMM4431-200358818
StARshRNA	Dharmacon Inc.	RMM4431-200393720

**Chemicals, peptides, and recombinant proteins**		

DMEM/F-12	Thermo Fischer Scientific	Cat#11330057
Collagenase D	Roche	Cat#11088858001
Dibutyryl cyclic AMP	Sigma-Aldrich	Cat#D0627
Cholesterol beads	Echelon Biosciences	Cat# P-BCHL
Dynabeads Protein G	Invitrogen	Cat#10003D
Fetal bovine serum	Thermo Fischer Scientific	Cat#A31607
Trypsin	Thermo Fischer Scientific	Cat#25200
Pen Strep	Thermo Fischer Scientific	Cat#15140
PBS	Sigma-Aldrich	Cat#P5368
X-tremeGENE HP DNA transfection reagent	Roche	Cat#06366236001
Plasmid Isolation kit	Bio-Rad	Cat#7326120
Opti-MEM media	Thermo Fischer Scientific	Cat#11058
hCG	Sigma-Aldrich	Cat# C8554
10X RIPA buffer	Cell Signaling Technology	Cat#9806S

**Critical commercial assays**		

Amplex Red Cholesterol Assay kit	Thermo Fischer Scientific	Cat#A12216
FSH ELISA kit	DRG International Inc.	Cat#EIA-1288
LH ELISA kit	DRG International Inc.	Cat#EIA-1289R
Progesterone ELISA kit	ENZO Lifesciences	Cat#ADI-900-011
Testosterone ELISA kit	DRG International Inc.	Cat#EIA-1559
Proteome profiler human phosphor-kinase array kit	R&D Systems	Cat#ARY003B

**Experimental models: Cell lines**		

MA-10 cell line	Generously provided by Dr. Zhenmin Lei, University of Louisville HSC, KY	
CRISPR/Cs9-Phb-MA-10 cells	Synthego, CA	

**Experimental models: Organisms/strains**		

Mouse: PHB-Tg	Developed in-house	Ref# 24
Mouse: Mutant PHB-Tg	Developed in-house	Ref# 30

**Software and algorithms**		

Biorender	https://biorender.com/	Biorender
BLAST	https://blast.ncbi.nlm.nih.gov/Blast.cgi?PAGE=Proteins	NCBI
GraphPad PRISM 6.2	https://www.graphpad.com	GraphPad software
Image Lab	https://www.bio-rad.com/en-ca/product/image-lab-software	ChemiDoc system Bio-Rad Laboratories)
Image*J*	https://imagej.nih.gov/ij/	

### Resource availability

#### Lead contact

Further information and requests for resources, including mouse models and cell lines, and reagents should be directed to the lead contact, Dr. Suresh Mishra (suresh.mishra@umanitoba.ca).

#### Materials availability

The study did not generate new unique reagents.

### Experimental model and subject details

#### Animal models

1. Trangenic mice expressing PHB1 from the Fabp-4 gene promoter
2. Transgenic mice expressing mPHB1 (Tyr114Phe) from the Fabp-4 gene promoter

The PHB1 and mPHB1 transgenic mice were developed by expressing human PHB and mPHB cDNA from the *Fabp-4* gene promoter. The cloning of PHB, as well as the development and phenotypic characterization of the PHB1 and mPHB1 transgenic mice have been reported previously (Ande et al., 2014, 2016a, 2016b). The male PHB1, mPHB1, and wild-type control mice were housed under a 12-h light-dark cycle at 22°C and were provided with normal chow (LabDiet, St. Louis, MO) and water *ad libitum*. All procedures were approved by the Animal Care and Use Committee of the University of Manitoba, Winnipeg, Canada (Protocol Approval #16-005, #20-008).

#### Primary Leydig cell (LC) isolation and culture

The testes were removed from the mice, sterilized using pre-chilled 70% ethanol, and washed three times with pre-chilled PBS. Epididymis, fat, and other connective tissues were removed from the testis samples using small scissors and forceps. The tunica albuginea was then dissected, and the testis samples were then placed into a 15 ml centrifuge tube containing a Dulbecco’s Modified Eagle Medium/nutrient mixture F-12 (DMEM/F-12) and 0.02% Collagenase D under constant agitation (90 rpm) for 30 min at 37°C, followed by an incubation undisturbed at room temperature for 10 min (Yamashita et al., 2011). Subsequently, the supernatant was filtered into a fresh 15 ml centrifuge tube containing 5 ml fresh DMEM/F-12 medium and was centrifuged at 200 x g for 4 min at room temperature. The pellet was washed twice with the fresh medium, and finally, the cells were resuspended in the DMEM/F-12 medium supplemented with 10% FBS for seeding (Yamashita et al., 2011). The cells were kept at 37°C with 5% CO_2_ in a humidified atmosphere.

#### Testis retrieval

First, the mice were anesthetized with 3% isoflurane and their blood was collected from the saphenous vein for hormonal analyses. Subsequently, the mice were euthanized by CO_2_ inhalation and the serum was stored at −20°C until analyzed. The abdomen area was sterilized with 70% alcohol and then a peritoneal incision was made using a sharp scalpel. The skin was opened anterior to the genitals to remove each of the testicles.

### Method details

#### Histological analysis

Testis samples from the 4-month-old PHB1, mPHB1, and wild-type mice were fixed in 4% buffered formaldehyde solution for 12 h, dehydrated, and embedded in a paraffin block. 5 μM-sections were stained with hematoxylin-eosin (Ande et al., 2014, 2016a, 2016b). The sections were analyzed under a light microscope and photomicrographs were captured using Evos (XL Core AMEX 1000, Invitrogen).

#### Electron microscopy

The transmission electron microscopy (TEM) of the testis was performed using a Philips CM10 at 80 kV at the Histomorphology & Ultrastructural Imaging Plat-form, in the Faculty of Health Sciences in the University of Manitoba. In brief, the testis samples were excised into small pieces (<1 mm^3^) and fixed with 3% glutaraldehyde in 0.1 M Sorensen’s buffer for 3 h. After fixation, the cells were resuspended in 5% sucrose in 0.1 M Sorensen’s buffer and then embedded in EPON^TM^ resin. TEM analysis was performed on ultra-thin sections (100 nm) and stained with uranyl acetate and counterstained with lead citrate (Ande et al., 2014).

#### MA-10 cell culture and transfection

The mouse Leydig cell line (MA-10 cells) were cultured in DMEM/F-12, supplemented with 10% FBS, and maintained at 37°C in a 5% CO_2_ humidified atmosphere (Yamashita et al., 2011). After 36 h in culture, cells were serum-starved for 6 h followed by transfection. The pCMV6-XL5 vector containing the human PHB1 clone was purchased from Origene Technologies and the cloning of the *m*-PHB1 cDNA construct has been reported previously (Ande et al., 2009). In brief, tyrosine 114 to phenylalanine (Tyr114Phe) mutant-PHB was made using site-directed mutagenesis kit (Stratagene, USA) following the manufacturer's instructions. The following primers were used for generating mutant-PHB (forward: 5′CAGCATCGGAGAGGACTTTGATGAGCGTGTGC -3′ and reverse: 5′-GCACACGCTCATCAAAGTCCTCTCCGATGCTG-3′). Authenticity of all constructs was confirmed by DNA sequencing. Glycerol stocks of PHB1 shRNA and StAR shRNA were purchased from Dharmacon Inc. The bacterial transformation, culture, and plasmid preparation were performed as described previously (Ande et al., 2009; Ande and Mishra, 2009) or following the manufacturer’s protocol. Cell transfection was performed using the X-treme GENE HP DNA transfection reagent according to the manufacturer’s protocol (Ande et al., 2009, 2012).

#### CRISPR/Cas9-Phb-MA-10 cells

CRISPR/Cas9-mediated Phb knockout MA-10 cells (CRISPR/Cas9-Phb-MA-10) was established using a custom service provided by Synthego (Menlo Park, CA). The editing efficiency after expansion of guide RNA (gRNA: UUACCAGGGACACGUCAUCC targeting Exon 5) transfected MA-10 KO cell pool was 79%. The site-specific targeting was confirmed using PCR and sequencing primers (F: GGGTTATAGCCATGAGTGTGCC and R: GTGTGCGGCAGACGAAACCT). Subsequently, knockout pool of MA-10 cells was subjected to serial dilution as per the manufacturer’s instruction and following standard protocol to establish clonal CRISPR/Cas9-Phb-MA-10 cell line.

#### Western blot

The MA-10 cells and primary LCs were lysed using an RIPA lysis buffer, and protein concentrations were quantified using Bradford’s assay. 20 μg of protein for each sample were run on SDS-PAGE (8–12%) gel, followed by a transfer to a PVDF membrane (Ande et al., 2009, 2012). Blots were then incubated in 5% non-fat milk made in TBST for an hour at room temperature, followed by an overnight incubation in the primary antibody at 4°C. Membranes were then washed in TBST for 3 × 10 min, followed by an hour of incubation in the secondary antibody at room temperature. Blots were washed again in TBST for 3 × 10 min and the ECL substrate was then used for imaging the blots using Imager (Bio-Rad).

#### Immunoprecipitation

The immunoprecipitation of PHB1, PHB2, StAR and P450scc was performed using a protein-specific antibody and a Dynabeads protein G suspension according to the manufacturer’s protocol. In brief, 10 μL of the protein-specific antibody (as applicable) were added to 500 μL of the cell lysate and incubated overnight on a rotating device at 4°C (Ande et al., 2009; Ande and Mishra, 2009)). At the end of the incubation, 20 μL of the Dynabeads protein G suspension was added to each tube and further incubated for 2 h. Subsequently, the pellets were washed 5 times in ice-cold PBS, resuspended in 2X loading buffer and analyzed by immunoblotting.

#### Cholesterol binding assay

An assay of the cholesterol beads was performed according to the manufacturer’s instructions. Briefly, to 500 μl of cell lysate, 20 μl of the cholesterol bead suspension was added and incubated at 4°C for 3 h on a rotating device. Subsequently, the pellets were washed 3 times with ice-cold PBS by centrifugation at 2000 rpm for 2 min at room temperature. Finally, the pellets were resuspended in 2X loading buffer and analyzed by immunoblotting using the anti-PHB1 and anti-PHB2 antibody.

#### Amplex red cholesterol assay

The cholesterol levels in Leydig cells were measured using an enzyme-coupled Amplex Red cholesterol assay kit as per the manufacturer’s instructions. This assay kit provides a simple fluorometric method for the sensitive quantitation of cholesterol using a fluorescence microplate reader or fluorimeter.

#### Proteome profiler human phospho-kinase array

The MA-10 cells were transfected with PHB1 and mPHB1 plasmid DNA for 36 h and treated with dibutyryl cAMP (db-cAMP, 0.5mM) for 2 h, and cell lysates were prepared post-transfection and treatment. The phosphorylation profile of the signaling pathways was detected by using the Proteome profiler human phospho-kinase array kit with 200 μg of protein samples. The assay was performed according to the manufacturer’s instructions and the images were captured using the Bio-Rad imaging system. Blot densities were analyzed by quantitative densitometry and each dot intensity was normalized to the reference dot’s intensities.

### Quantification and statistical analysis

Quantification of band densities, lipid droplets, mitochondrial numbers, and lipid droplet areas were performed using ImageJ software (https://imagej.nih.gov/ij/). GraphPad Prism 6.2 software was used for the statistical analysis in all experiments. For comparisons between two groups, a two-tailed student’s t test was performed and for multiple comparisons, an analysis of variance (ANOVA) with Dunnett’s test was used (Ande et al., 2014, 2016a, 2016b). A p value of < 0.05 was considered statistically significant in all cases. The graphs represent means, and error bars indicate the standard error of the mean (SEM). All experiments were repeated for at least 3 times and p values are reported in the respective figure legends.

## Data Availability

All data produced in this study are included in the published article and its supplemental information, or are available from the lead contact upon request. This paper does not report original code. Any additional information required to reanalyze the data reported in this paper is available from the lead contact upon request.

## References

[bib1] Ande S.R., Mishra S. (2009). Prohibitin interacts with phosphatidylinositol 3,4,5-triphosphate (PIP3) and modulates insulin signaling. Biochem. Biophys. Res. Commun..

[bib2] Ande S.R., Gu Y., Nyomba B.L., Mishra S. (2009). Insulin induced phosphorylation of prohibitin at tyrosine 114 recruits Shp1. Biochim. Biophys. Acta.

[bib3] Ande S.R., Xu Z., Gu Y., Mishra S. (2012). Prohibitin has an important role in adipocyte differentiation. Int. J. Obes. (Lond.).

[bib4] Ande S.R., Nguyen K.H., Padilla-Meier G.P., Wahida W., Nyomba B.L., Mishra S. (2014). Prohibitin overexpression in adipocytes induces mitochondrial biogenesis, leads to obesity development, and affects glucose homeostasis in a sex-specific manner. Diabetes.

[bib5] Ande S.R., Nguyen K.H., Nyomba B.L.G., Mishra S. (2016). Prohibitin in adipose and immune functions. Trends Endocrinol. Metab..

[bib6] Ande S.R., Nguyen K.H., Padilla-Meier G.P., Nyomba B.L., Mishra S. (2016). Expression of a mutant prohibitin from the aP2 gene promoter leads to obesity-linked tumor development in insulin resistance-dependent manner. Oncogene.

[bib7] Anderson C.J., Kahl A., Fruitman H., Qian L., Zhou P., Manfredi G., Iadecola C. (2020). Prohibitin levels regulate OMA1 activity and turnover in neurons. Cell Death Differ..

[bib8] Ascoli M. (1981). Characterization of several clonal lines of cultured Leydig tumor cells: gonadotropin receptors and steroidogenic responses. Endocrinology.

[bib9] Bose H.S., Lingappa V.R., Miller W.L. (2002). Rapid regulation of steroidogenesis by mitochondrial protein import. Nature.

[bib10] Browman D.T., Resek M.E., Zajchowski L.D., Robbins S.M. (2006). Erlin-1 and erlin-2 are novel members of the prohibitin family of proteins that define lipid-raft-like domains of the ER. J. Cell Sci..

[bib11] Castillo A.F., Orlando U., Helfenberger K.E., Poderoso C., Podesta E.J. (2015). The role of mitochondrial fusion and StAR phosphorylation in the regulation of StAR activity and steroidogenesis. Mol. Cell. Endocrinol..

[bib12] Chien Y., Cheng W.C., Wu M.R., Jiang S.T., Shen C.K., Chung B.C. (2003). Misregulated progesterone secretion and impaired pregnancy in Cyp11a1 transgenic mice. Biol. Reprod..

[bib13] Chowdhury I., Xu W., Stiles J.K., Zeleznik A., Yao X., Matthews R., Thomas K., Thompson W.E. (2007). Apoptosis of rat granulosa cells after staurosporine and serum withdrawal is suppressed by adenovirus-directed overexpression of prohibitin. Endocrinology.

[bib14] Chowdhury I., Thompson W.E., Welch C., Thomas K., Matthews R. (2013). Prohibitin (PHB) inhibits apoptosis in rat granulosa cells (GCs) through the extracellular signal-regulated kinase 1/2 (ERK1/2) and the Bcl family of proteins. Apoptosis.

[bib15] Chowdhury I., Thomas K., Zeleznik A., Thompson W.E. (2016). Prohibitin regulates the FSH signaling pathway in rat granulosa cell differentiation. J. Mol. Endocrinol..

[bib16] Dong P., Flores J., Pelton K., Solomon K.R. (2010). Prohibitin is a cholesterol-sensitive regulator of cell cycle transit. J. Cell. Biochem..

[bib17] Duarte A., Poderoso C., Cooke M., Soria G., Cornejo Maciel F., Gottifredi V., Podestá E.J. (2012). Mitochondrial fusion is essential for steroid biosynthesis. PLoS One.

[bib18] Duarte A., Castillo A.F., Podestá E.J., Poderoso C. (2014). Mitochondrial fusion and ERK activity regulate steroidogenic acute regulatory protein localization in mitochondria. PLoS One.

[bib19] Elustondo P., Martin L.A., Karten B. (2017). Mitochondrial cholesterol transport. Biochim. Biophys. Acta.

[bib20] Faulkner J.L., Belin de Chantemèle E.J. (2019). Sex hormones, aging and cardiometabolic syndrome. Biol. Sex Differ..

[bib21] Gallolu Kankanamalage S., Lee A.Y., Wichaidit C., Lorente-Rodriguez A., Shah A.M., Stippec S., Whitehurst A.W., Cobb M.H. (2016). Multistep regulation of autophagy by WNK1. Proc. Natl. Acad. Sci. U S A.

[bib22] Gao F., Li G., Liu C., Gao H., Wang H., Liu W., Chen M., Shang Y., Wang L., Shi J. (2018). Autophagy regulates testosterone synthesis by facilitating cholesterol uptake in Leydig cells. J. Cell Biol..

[bib23] Huber T.B., Schermer B., Müller R.U., Höhne M., Bartram M., Calixto A., Hagmann H., Reinhardt C., Koos F., Kunzelmann K. (2006). Podocin and MEC-2 bind cholesterol to regulate the activity of associated ion channels. Proc. Natl. Acad. Sci. U S A.

[bib24] Ishii T., Hasegawa T., Pai C.I., Yvgi-Ohana N., Timberg R., Zhao L., Majdic G., Chung B.C., Orly J., Parker K.L. (2002). The roles of circulating high-density lipoproteins and trophic hormones in the phenotype of knockout mice lacking the steroidogenic acute regulatory protein. Mol. Endocrinol..

[bib25] Kang T., Lu W., Xu W., Anderson L., Bacanamwo M., Thompson W., Chen Y.E., Liu D. (2013). MicroRNA-27 (miR-27) targets prohibitin and impairs adipocyte differentiation and mitochondrial function in human adipose-derived stem cells. J. Biol. Chem..

[bib26] Kaprara A., Huhtaniemi I.T. (2018). The hypothalamus-pituitary-gonad axis: tales of mice and men. Metabolism.

[bib27] Kim D.K., Kim H.S., Kim A.R., Jang G.H., Kim H.W., Park Y.H., Kim B., Park Y.M., Beaven M.A., Kim Y.M., Choi W.S. (2013). The scaffold protein prohibitin is required for antigen-stimulated signaling in mast cells. Sci. Signal..

[bib28] Kusminski C.M., Holland W.L., Sun K., Park J., Spurgin S.B., Lin Y., Askew G.R., Simcox J.A., McClain D.A., Li C., Scherer P.E. (2012). MitoNEET-driven alterations in adipocyte mitochondrial activity reveal a crucial adaptive process that preserves insulin sensitivity in obesity. Nat. Med..

[bib29] Lei Z.M., Mishra S., Zou W., Xu B., Foltz M., Li X., Rao C.V. (2001). Targeted disruption of luteinizing hormone/human chorionic gonadotropin receptor gene. Mol. Endocrinol..

[bib30] Liu K., Czaja M.J. (2013). Regulation of lipid stores and metabolism by lipophagy. Cell Death Differ..

[bib31] Ma Y., Zhou Y., Zhu Y.C., Wang S.Q., Ping P., Chen X.F. (2018). Lipophagy contributes to testosterone biosynthesis in male rat Leydig cells. Endocrinology.

[bib32] Medar M.L.J., Marinkovic D.Z., Kojic Z., Becin A.P., Starovlah I.M., Kravic-Stevovic T., Andric S.A., Kostic T.S. (2021). Dependence of Leydig cell’s mitochondrial physiology on luteinizing hormone signaling. Life.

[bib33] Merkwirth C., Dargazanli S., Tatsuta T., Geimer S., Löwer B., Wunderlich F.T., von Kleist-Retzow J.C., Waisman A., Westermann B., Langer T. (2008). Prohibitins control cell proliferation and apoptosis by regulating OPA1-dependent cristae morphogenesis in mitochondria. Genes Dev..

[bib34] Midzak A., Papadopoulos V. (2016). Adrenal mitochondria and steroidogenesis: from individual proteins to functional protein assemblies. Front. Endocrinol. (Lausanne).

[bib35] Midzak A., Akula N., Lecanu L., Papadopoulos V. (2011). Novel androstenetriol interacts with the mitochondrial translocator protein and controls steroidogenesis. J. Biol. Chem..

[bib36] Miller W.L. (1997). Congenital lipoid adrenal hyperplasia: the human gene knockout of the steroidogenic acute regulatory protein. J. Mol. Endocrinol..

[bib37] Miller W.L., Auchus R.J. (2011). The molecular biology, biochemistry, and physiology of human steroidogenesis and its disorders. Endocr. Rev..

[bib38] Monté D., DeWitte F., Hum D.W. (1998). Regulation of the human P450scc gene by steroidogenic factor 1 is mediated by CBP/p300. J. Biol. Chem..

[bib39] O’Hara L., McInnes K., Simitsidellis I., Morgan S., Atanassova N., Slowikowska-Hilczer J., Kula K., Szarras-Czapnik M., Milne L., Mitchell R.T., Smith L.B. (2015). Autocrine androgen action is essential for Leydig cell maturation and function, and protects against late-onset Leydig cell apoptosis in both mice and men. FASEB J..

[bib40] Osman C., Haag M., Potting C., Rodenfels J., Dip P.V., Wieland F.T., Brügger B., Westermann B., Langer T. (2009). The genetic interactome of prohibitins: coordinated control of cardiolipin and phosphatidylethanolamine by conserved regulators in mitochondria. J. Cell Biol..

[bib41] Osman C., Merkwirth C., Langer T. (2009). Prohibitins and the functional compartmentalization of mitochondrial membranes. J. Cell Sci..

[bib42] Oyola M.G., Handa R.J. (2017). Hypothalamic-pituitary-adrenal and hypothalamic-pituitary-gonadal axes: sex differences in regulation of stress responsivity. Stress.

[bib43] Park J.E., Kim Y.J., Lee S.G., Kim J.Y., Chung J.Y., Jeong S.Y., Koh H., Yun J., Park H.T., Yoo Y.H., Kim J.M. (2019). Drp1 phosphorylation is indispensable for steroidogenesis in Leydig cells. Endocrinology.

[bib44] Rajalingam K., Wunder C., Brinkmann V., Churin Y., Hekman M., Sievers C., Rapp U.R., Rudel T. (2005). Prohibitin is required for Ras-induced Raf-MEK-ERK activation and epithelial cell migration. Nat. Cell Biol..

[bib45] Reichman D., Rosenwaks Z. (2017). The impact of genetic steroid disorders on human fertility. J. Steroid Biochem. Mol. Biol..

[bib46] Rodriguez-Gonzalez A., Cyrus K., Salcius M., Kim K., Crews C.M., Deshaies R.J., Sakamoto K.M. (2008). Targeting steroid hormone receptors for ubiquitination and degradation in breast and prostate cancer. Oncogene.

[bib47] Romanowski M.J., Soccio R.E., Breslow J.L., Burley S.K. (2002). Crystal structure of the Mus musculus cholesterol-regulated START protein 4 (StarD4) containing a StAR-related lipid transfer domain. Proc. Natl. Acad. Sci. U S A.

[bib48] Rone M.B., Fan J., Papadopoulos V. (2009). Cholesterol transport in steroid biosynthesis: role of protein-protein interactions and implications in disease states. Biochim. Biophys. Acta.

[bib49] Steglich G., Neupert W., Langer T. (1999). Prohibitins regulate membrane protein degradation by the m-AAA protease in mitochondria. Mol. Cell. Biol..

[bib50] Tatsuta T., Model K., Langer T. (2005). Formation of membrane-bound ring complexes by prohibitins in mitochondria. Mol. Biol. Cell.

[bib51] Thompson W.E., Sanbuissho A., Lee G.Y., Anderson E. (1997). Steroidogenic acute regulatory (StAR) protein (p25) and prohibitin (p28) from cultured rat ovarian granulosa cells. J. Reprod. Fertil..

[bib52] Thompson W.E., Asselin E., Branch A., Stiles J.K., Sutovsky P., Lai L., Im G.S., Prather R.S., Isom S.C., Rucker E., Tsang B.K. (2004). Regulation of prohibitin expression during follicular development and atresia in the mammalian ovary. Biol. Reprod..

[bib53] Tsujishita Y., Hurley J.H. (2000). Structure and lipid transport mechanism of a StAR-related domain. Nat. Struct. Biol..

[bib54] Wang Q., Leader A., Tsang B.K. (2013). Follicular stage-dependent regulation of apoptosis and steroidogenesis by prohibitin in rat granulosa cells. J. Ovarian Res..

[bib55] Wang Q., Leader A., Tsang B.K. (2013). Inhibitory roles of prohibitin and chemerin in FSH-induced rat granulosa cell steroidogenesis. Endocrinology.

[bib56] Wasilewski M., Semenzato M., Rafelski S.M., Robbins J., Bakardjiev A.I., Scorrano L. (2012). Optic atrophy 1-dependent mitochondrial remodeling controls steroidogenesis in trophoblasts. Curr. Biol..

[bib57] Wei Y., Chiang W.C., Sumpter R., Mishra P., Levine B. (2017). Prohibitin 2 is an inner mitochondrial membrane mitophagy receptor. Cell.

[bib58] Wood C.L., Lane L.C., Cheetham T. (2019). Puberty: normal physiology (brief overview). Best Pract. Res. Clin. Endocrinol. Metab..

[bib59] Yamashita S., Tai P., Charron J., Ko C., Ascoli M. (2011). The Leydig cell MEK/ERK pathway is critical for maintaining a functional population of adult Leydig cells and for fertility. Mol. Endocrinol..

[bib60] Yang G., Xu H., Li Z., Li F. (2014). Interactions of caveolin-1 scaffolding and intramembrane regions containing a CRAC motif with cholesterol in lipid bilayers. Biochim. Biophys. Acta.

